# Reducing Information Pollution in the Internet Age

**Published:** 2006-12-15

**Authors:** David E Nelson

**Affiliations:** Health Communications Branch, Office on Smoking and Health, National Center for Chronic Disease Prevention and Health Promotion, Centers for Disease Control and Prevention

I was recently asked by an outside organization to provide comments about a proposed application for the organization's Web site. The individuals involved had obviously spent time and money and were far along in site development. They wanted feedback about design features such as layout, use of maps, and alternative views of the site. The developers assumed that the information they were providing was important and would help state and local public health professionals do their planning. Those of us participating in the Web application review pointed out in a tactful way that the developers seemed to have little understanding of the public health community and its needs and that this application would be of little interest to public health practitioners and planners. I doubt I will be asked to participate in future reviews of Web applications for this organization.

This story demonstrates the problems that communication technology advancements can cause for public health and other fields. Individuals and organizations can now provide large amounts of information at low cost by using a myriad of information tools (1). These advances, while exciting, are seductive and can cause people to forget some communication fundamentals, such as understanding intended audiences and how they seek, assess, and use information.

People who remember communication fundamentals have decided advantages in reaching audiences with their messages. Public health officials who fail to put communication basics into practice will be unlikely to improve public health. Instead, they will contribute to the growing problem of information overload or *data smog* (2).

Public health organizations that use the Internet to convey information can easily fall into the trap of providing on their sites as much information as possible for potential users. This *kitchen sink* approach may seem attractive at first glance (3,4). After all, given the potential worldwide size of the Internet audience, somebody somewhere might find any individual piece of information useful. This approach is highly democratic, fits well within American cultural values and freedom of choice, and has the added advantage of reducing the possibility of criticism from management or colleagues because information they desire is not available on a site. Perhaps most importantly, it means avoiding having to decide, or prioritize, what information to include or exclude on a Web site.

Simply providing more and more health information may not lead to increased information use or desired public health improvements. A good example is the effort to make information about health care performance by hospitals, health plans, and individual providers widely available to the public (5,6). The rationale for publicizing health care quality information, or *report cards*, was a belief that doing so would facilitate informed choice by consumers and stimulate improvements in the quality of health care (7). Despite widespread availability of this information, there is little evidence that health care consumers have sought such information on Web sites or that this information has influenced their health care decisions (5-7). The lesson here is that providers of health care information need to know their audiences before creating and distributing information.

Web sites are now a critical source of health information. An estimated 64% of U.S. adults have searched for health information online for themselves or others within the previous 12 months (8). Well-designed Web sites allow health information seekers to find what they need quickly. In contrast, poorly designed sites result in users becoming frustrated and impatient and often lead them to search elsewhere (9). Many developers of and contributors to public health Web sites have little understanding of Internet audiences — particularly how they search for and assess information. Fortunately, research in psychology, communication, and related disciplines is providing insights about improving Web sites.

The importance of how audiences perceive the credibility of information sources cannot be overestimated (10). People rapidly assess the believability of information based on how much they trust the individual or organization providing it. This process is referred to in psychology as the *expert heuristic* (11). Public health departments and other health-related organizations with high ethical standards, no perceived conflicts of interest, and high-quality, evidence-based information are normally considered credible information sources. Public trust is invaluable because credibility, once lost, is difficult to regain. It is important that public health professionals and organizations act ethically to maintain trust.

Determining which audiences need to be reached and the communication channels they prefer are crucial early steps in designing appropriate communication strategies. Target audiences for many public health messages — people with low incomes, those with low education levels, and the elderly — are less likely to use Web sites than other demographic groups (8). Public health professionals need to consider other communication channels to reach these audiences.

Segmenting Internet users according to their information needs and preferences is another important way to make Web sites more relevant to particular groups. During the process of redesigning its Web site, the Office on Smoking and Health (OSH) at the Centers for Disease Control and Prevention (CDC) discovered it had seven major audiences with somewhat different information needs: 1) public health professionals such as state program managers, 2) researchers and scientists, 3) CDC partner organizations such as the Campaign for Tobacco-Free Kids, 4) policy advocates, 5) public health educators, 6) media professionals, and 7) the general public.

Understanding the type of information audiences need enables an organization to develop and maintain a Web site with materials at appropriate literacy levels and organized in ways to meet audience expectations (12). OSH, for example, discovered that its audiences preferred information organized by tobacco topics (e.g., secondhand smoke, smoking cessation) rather than by other classifications such as CDC organizational areas or audience types.

It is also important to know what related information is offered on the Web sites of other organizations. This learning process can lead to the discovery of effective information materials or other products that have already been developed, and there is little point in duplicating efforts. Providing effective information that is complementary to what is offered on other sites is helpful, and having links to trusted sites is valuable for site users. Through the process of searching and linking public health groups can identify interorganizational collaborative opportunities.

Readers of this editorial, along with millions of other Internet users, probably rely on search engines, like Google and others, to locate Web links. Search engines have revolutionized how people access Internet information. It is invaluable to conduct search engine research to determine whether, and where, a site appears when common key words are entered. The large amount of information a search engine provides results in a tendency for people to scan Web pages rapidly. If an organization's site is not listed within the first few pages of a search, the organization may need to make changes to move the site higher on search engine lists. Public health officials need to make others aware of good Web site information through the dreaded *M* word — marketing. Letting audiences know where information is available on the Web is increasingly essential because of the large number of sources available online.

People are exposed to hundreds of media messages each day, mostly in the form of advertising. The human brain is remarkably adept at blocking information a person does not consider relevant (11). A person can hone in quickly on information that is salient, which explains the tendency of people to rapidly scan Web sites. Information seekers tend to work no harder than they need to in order to find relevant information (i.e., they are *cognitive misers*). Once they determine that what they have is good enough, they rarely search further. This tendency, first described in the 1950s, is referred to as *satisficing* (11) and is especially common among people who are time-pressured, such as journalists and health care providers. People exposed to excessive information tend to rely on simple rules such as selecting the first item from a lengthy list.

These tendencies to scan quickly for salient information and satisfice mean that Web sites must make it easy for information seekers to find what they want quickly (9). Materials need to be organized in ways a site user thinks about a topic. Text should be kept to a minimum, and pages should contain sufficient open space to seem uncluttered. Few mouse clicks should be required to locate useful information, and design layout (e.g., colors, logos, background information) should be consistent. Usability testing with intended audience members can help uncover layout problems (9).

The tendencies of Internet users to scan quickly for salient information help explain why providing too much material on a Web site (the kitchen sink approach) is counterproductive. Barry Schwartz, in a recent review of psychology literature, refers to the effect of giving too many options as the *paradox of choice* (4) (i.e., providing too many options reduces the ability of people to make better decisions). Understanding how audiences seek and process information from Web sites can lead to better decisions about what information to provide and how to organize it effectively. Too much information on a site has another downside for organizations because resources and extensive efforts are required to update information regularly.

The Coordinating Center for Health Promotion (CoCHP) at CDC is involved in developing and improving cross-cutting efforts in public health to reduce obesity and advance newborn screening initiatives (13). Designing a useful Web site based on effective communication concepts will be important for the health education function of CoCHP's new site. The new Web site should make it easy for users to find what they need and, if done well, will provide an important public health service rather than adding to Internet information pollution.
